# Chicago Medical Society

**Published:** 1884-03

**Authors:** 


CHICAGO MEDICAL SOCIETY.

The regular semi-monthly session of this Society, January 21st, 1884, was held in the parlors of the "Pacific," and was of unusual interest.

Dr. Charles G. Davis read an exhaustive paper on our indigenous remedy, "Gelseminum Sempervirens." His preliminary remarks and the principal points in the paper were as follows:

The present advanced state of medical science is due in a great measure, if not entirely, to the recorded experience and observation of thinking men. If this be true, would it not be well if all physicians, engaged in the active practice of their profession, made a special endeavor to observe more closely, and record more accurately, the various symptoms manifested by the action of medicine on the human system. A neglect of this on the part of many physicians has given rise to what we may properly term the "school of skepticism." They disbelieve entirely in the efficacy of medicine to relieve disease, and often, while manifesting a sublime indifference to the appeals of the poor sufferer before them to be relieved, they go into ecstacies over the pathology of the case, and are plethoric with theories in regard to the ultimate nerve cells or molecules wherein the disturbing action arises.

Be it understood, I cast no reflection upon scientific investigation in these directions. But the true office of the physician is to prevent, cure, or alleviate disease, and in proportion as he fails in this, he fails to fulfil his mission.

To-night I lay before you my recorded experience in regard to the action of a remedy which for a number of years has been under my observation.

Gelseminum Sempervirens commonly known as the wild, yellow, or Carolina jasmine, was first described in 1640, by John Parkinson, who grew it in his garden. (See the U. S. and other dispensatories for its properties, further description, etc., etc.)

The writer is thoroughly convinced there is no article of equal merit in the materia medica which has received so little notice, and concerning which so little is known; and the objects of the paper, as set forth, were to give a synopsis of the different theories concerning its mode of action with his own testimony added, especially in regard to its therapeutical application. This which is here synopsized from the paper based upon the personal experience of the author. The following anecdotemay not be devoid of interest. The manner in which, according to the chronicler of the story, the remedy was discovered, may have been, however, an accident.

A Southern gentleman, who was sick with a fever, sent his colored servant into the gardent to obtain the roots of a plant which he indicated, in order that he might prepare a decoction for himself, and alleviate his disease. The servant made a mistake, and obtained instead the roots of the yellow jasmine. The tea was drunk, however, and the patient, breaking out into a profuse perspiration and recovering soon from the fever, made inquiry as to what he had taken, and was so delighted with the result that he immediately recommended it to others who were similarly afflicted.

However this may be, gelseminum owes its medicinal action to presence of an alkaloid, its active principle, viz.:—gelsemina or gelsemia; and to Prof. T. G. Wormly undoubtedly belongs the credit of having first isolated the alkaloid, who also demonstrated the presence of a new organic acid, which he designated gelseminic acid. By his method, sixteen ounces of the fluid extract of gelseminum yielded about two grains and a quarter of the of pure gelseminic acid, which is colorless, odorless, mostly tasteless, crystallizes in groups or tufts of delicate needles, has strongly acid properties, neutralizing bases, and uniting with them to form salts which are generally sparingly soluble in water.

Gelsemia may be extracted from the concentrated extract from which gelseminic acid has been extracted, by a process for a minute description of which see the various dispensatories, materiæ medicæ, works on chemistry, etc., etc. In general, the author stated, the amount of the alkaloid found in any given quantity of the fluid extract (the only officinal preparation used) approximately existed to the extent of one grain to two and a half fluid ounces of the fluid extract.

*Physiological actions.*—Gelsemia is so intensely bitter that its presence to the extent of one-thousandth part by weight in solution can be readily detected, and when taken into the system it is rapidly diffused into the blood.

In regard to its action on the human system, and also the lower animals, the following has been deduced from the author's voluminous paper: When administered in small quantities, sufficient to produce a sensible effect, it has a mild, soothing tendeney over the nervous system, manifested by mental quietude; a tendency to drooping of the eyelids; slight muscular relaxation; slowing of the pulse and slight dilatation of the pupil, though Prof. Ringer says this dilatation is preceded by contraction of the pupil. When the dose is much increased, the symptoms are all intensified, and we see manifested double vision; giddiness; pain in the forehead; increased paralysis of the levator palpebræ muscles, causing drooping of the eyelids; labored respiration, from partial paralysis of the respiratory muscles; muscular weakness; and general sensibility is much reduced. If the dose is still further increased, the symptoms are still more intensified; the action of the heart becomes weak and intermittent; vision becomes entirely lost; pupils widely dilated, not responding to the action of light; consciousness may be retained until near the period of death, when it is lost from the carbonic acid poisoning resulting from incomplete respiration.

The action of the heart continues for some moments after respiration has ceased. From these symptoms it is evident that the prime action of gelsemia is upon the nervous system, and its action is such as to warrant us in classifying it as a cerebro-spinal depressant, acting principally upon the motor centers, although some doubt has been expressed that its primary action is upon the peripheral extremities of the motor nerves. Ringer at one time entertained this view of its action primarily upon the extremities of the motor sensitive nerves. In cold-blooded animals its first effect is on the sensory center, while in warm-blooded animals the motor centers are first impressed. The reader of the paper considered this fact to be of great importance, viz.: that the remedy lowers the temperature, which he had verified in many febrile and inflammatory diseases. The theory of its being an efficient antidote to poisoning by strychnia was, according to the views expressed by the writer, disproven, as he illustrated by experiments on dogs, and the contrary effect, he was satisfied to announce, was the result—that it facilitated or increased the action of strychnia. Two dogs were selected, weighing ten pounds each, to both was administered at the same time one-fifth of a grain of sulphate of strychnia hypodermically. Five minutes elapsed, and one-eighth of a grain of the alkaloid gelsemia in the form of a sulphate was given to one of the dogs. In ten minutes convulsions appeared in the animal, while the effects were not noticed in the dog that received only the strychnia until the end of thirteen minutes. In thirty minutes the animal that had received both poisons was dead, while the other lived forty minutes, and then too it was noticeable in the experiment that in the animal receiving both the strychnia and gelsemia, the convulsions which appeared earlier were far more violent and continuous. This experiment was repeated a number of times with almost unvarying results. As antagonistic to and incompatible with the effects of gelsemium, we may rely upon alcoholic and diffusible stimulants, and, as an illustration, a case was cited that the author treated three years ago. J. B., æt. 45, had been afflicted with muscular rheumatism for a number of years, was given five drops of the f. e. gelseminum three times a day, with instructions to increase the dose one drop every day. In time a dose of twenty drops was taken every day, and not seeing any of the usual effects of such a dose I requested him to change his druggist. (It is well known that there is occasionally a druggist who is not infallible; and the drugs he offers for sale to a willing public, for which he often feels desirous also to prescribe are often to be placed in the same category). In two hours after acting upon this advice the patient was brought back to my office by a worthy officer who found the gentleman staggering on the street, and supposed him intoxicated, as he showed to a very marked degree the effects of the remedy. A tablespoonful of brandy was immediately administered. In fifteen minutes he was much improved. The same amount of the stimulant was repeated twice during an hour, at the expiration of which time the symptoms had entirely disappeared. Thiscase serves to illustrate the antidotal action of alcoholic stimulants to the poisonous action of gelseminum; also, what is true of many other of the fluid extracts—their great variation in medicinal action.

*Therapy.*—In its native southern region it has for many years had an extensive reputation as a remedy of great value in the treatment of autumnal malarial fevers. While its efficacy in this direction may have been overestimated, yet its beneficial effects in malarial fevers are such as to merit our careful consideration. After an observation of a number of years, and administering the remedy probably to many hundreds of cases, I have arrived at the following conclusions:

First. Gelsemium is not a direct antidote, either chemically or physiologically, to malaria.

Second. It does have a wonderful effect over the disease, owing to the relaxing influence it brings to bear upon the system, thereby increasing excretion and assisting to eliminate morbific matters.

Third. It may be regarded as the great adjuvant of the salts of cinchona, and in this direction, so far as malaria is concerned, lies its chief merit.

The following typical case, of which the principal points only are here given, illustrates this action.

A sign painter of bilious temperament, sallow complexion and melancholy expression of countenance, had ague at intervals for three years, of which the smallest amount of quinine, or any other of the salts of cinchona produced unusually severe cerebral symptoms. Was called to see him while he was seized with a terrible chill, simulating the congestive form. He refused the treatment that was suggested, and in forty-eight hours he was again prostrated with another more severe attack. When called to see him again he was partially unconscious in a congestive chill. After three hours' hard work, with dilligent use of stimulants, external heat, etc., reaction began. During the febrile stage, the f. e. of the gelsemium was given, freely reducing the temperature, and producing symptoms denoting its other effects. After eighteen hours had elapsed ten grains of quinine were given every two hours till 120 grains had been given. Not a symptom of tinnitus aurium was manifested, nor any other unpleasant symptom was noticeable. There has been no return of the disease from that day to this so far as is known. This is only one of a number of cases where the remedy was given to prepare the system for the action of quinine.

In Convulsive Diseases, particularly those where the spasm is dependent upon reflex irritation, this remedy satisfactorily fulfils the indications, especially in the various diseases of childhood where convulsions are manifested. The following illustration may not be devoid of interest.

Willie S—, aged 8 months, at supper partook of milk and mashed potatoes. The child was soon seized with convulsions, because the stomach failed to digest its contents. In three hours time there had been twenty convulsions according to the statements of the attendants. Upon arriving, two drops of the f. e. were immediately given to the child, and in ten minutes two drops more.

Then ten drops were prepared in four ounces of water, and a teaspoonful of this was given every hour for twelve hours longer. No more convulsions appeared, and the next day the child was convalescent. No other remedy has such a satisfactory effect on the nervous system of a child during the period of dentition, while the entire system is disturbed from irritation of the distal extremities of the fifth pair of nerves.

As a remedy for *meningeal inflammation* of the brain and spinal cord, its efficacy in this class of cases the reader illustrated by citing a case selected from a number of similar ones that had from time to time been under his observation, and the large amount of the remedy that may be borne when the nerve centers are excited. When called to the bedside, the case (that of a young man) had already progressed four days, his temperature was high, pulse rapid and small, tongue dry, mind wandering, and there was severe spinal tenderness; the slightest disturbance in the room, or jar of the bed resulted in severe convulsions, which amounted to opisthotonos, occurring once or twice every hour. Bromides in large doses had already been given. The f. e. of gelsemium in five drop doses was then administered every hour for three hours, at which time another convulsion came on. Ten drops of the remedy were then ordered every hour. In three hours there appeared slight symptoms of another convulsion. The dose was increased to twelve drops, and gradually to fifteen drops, given as often, before there was entire relaxation and disappearance of all convulsive action. This dose was maintained for several hours before any toxic symptoms began to be manifest. The patient was then continued under the influence of small doses for a week longer, and later he recovered. Gelsemium has been lauded as a remedy for tetanus, but the author is convinced that it is of but little use in this condition. It has no effect within itself over epilepsy, but added to bromides, a desired result may be obtained more immediately and permanently in controlling cases of epilepsy confined to children.

The writer doubts its efficacy in cases of tic-doloreux or neuralgia, and thought it quite possible that the successful case s treated by the remedy by others were not tic, but neuralgia, resulting from cold or some other excentric causes producing congestion. Given in pneumonitis and pleuriti-, it is beneficial, and will lessen or retard the inflammatory conditions by lowering the temperature, lessening the pulse rate, and it has a soothing effect over the respiratory functions. It has a marked effect over the mucous surfaces, in gastro-intestinal catarrh, dysentery, and inflammatory conditions of the urinary organs. In rattlesnake bites, no other remedy yielded such results, while the author was living in Kansas, some 12 years ago, where he treated a number of these cases. The local inflammation and pain rapidly subsided, and convulsions ceased after giving a few doses.

Favorable reports from several physicians to whom the remedy was suggested verify this. The remedy is given internally and applied locally, and in these cases its action, doubtless, is entirely physiological and not chemical.

*In diseases of women*, of which a variety were enumerated, he referred the application of the remedy to pelvic disorders and their attendant nervous disturbances, and it applies with equal force to both the acute and chronic states, for it possesses a relaxing effect over the uterus and its appendages.

In the first stage of labor, where there may be present a rigid and unyielding os, a similar administration will produce many times a most happy effect. In many forms of dysmenorrhœa its beneficial effects are equally noticeable. The author pictured many typical cases classified under the head of "nervous diseases;" "long continued congestions of the pelvic visc era;" "various phases of hyperæmia and anæmia of the spinal cord;" "hysteria;" "spinal irritation;" "neurasthenia," etc., etc., that were treated by all known therapeutical measures, including "local treatment," and while he did not claim it to be a never-failing remedy—a panacea—for all these conditions, he held that a large number of female diseases could be relieved by this remedy. In using it for a number of years, continued the writer, he was prepared to positively assert that gelseminum is a stimulant or tonic to the cerebro-spinal system when long continued in small quantities.

Three chronic cases were cited of local troubles (ulceration) with serious nervous disturbance, in which all were relieved, in fact, recovered, by taking minute doses of the remedy. The first was where the patient had been treated for endocervicitis, retroflexion, retroversion, ovaritis, peri-uterine trouble, etc., etc.; menstruation had ceased. She was 35 years of age ; all the functions were gradually restored, and in eighteen months she gave birth to a perfectly healthy child, since which time her health has been perfect.

Case Second was a remarkable one, a patient aged 45, with a decidedly neurotic history. There was hemi-anæsthesia, but this was not due to any cerebral lesion ; there was ovarian tenderness ; bowels constipated ; frequent mictruition ; bearing-down pains ; uterus enlarged and retroverted. She suffered severely with dysmenorrhœa, and sometimes the menses did not appear at all. She was approaching the "turn of life ;" she had been married 20 years, but had never been pregnant. Her improvement was gradual and continuous by taking small doses of gelseminum, and in six months she appeared entirely well, menstruation being regular and normal. In three months longer she called again, for she was afflicted with nausea, and felt, as she expressed it, "so very strangely." Could she have conceived? Yes, possibly ; and in due time "the wife and son were doing well," was the message sent me. Thus the pathological condition was removed, menstruation had returned, and pregnancy occurred at the age of 45 years.

The last case, that of a lady, aged 46, who consulted the writer some four years ago. Her case presented a number of interesting features. For eight years she had been in a "decline," and she became reduced in weight to seventy-five and one-half pounds. During the past five years she had been treated for typhoid fever, which once had attacked her and continued for five weeks. She has been treated also for sciatica, bronchitis, ulceration of the intestinal canal, disease of the pylorus, cirrhosis of the liver, diseased kidneys, hyperæmia of the brain, and the various flexions, versions, and inflammation of the uterus. My diagnosis was given her, facetiously perhaps, by saying that "she was sick." There was a general failure of nutrition, restlessness; frequent attacks of neuralgia; depression of spirits; spinal tenderness; enfeebled digestion, and back-ache. Her menstruation had been very scanty and irregular for two or three years, and had not appeared for seven months. The gelseminum was given to her in minute doses. She began to improve almost immediately. At the end of the third month menstruation reappeared, much to our surprise, (her's as well as my own). In four months she gained 20 pounds, and in a few weeks longer her weight was increased 35 pounds, as was also her strength and general health, and menstruation still continues at this date, she having attained the age of almost 50 years.

As a remedy for noctural emissions, it was simply referred to in the paper; and given alone, not much benefit need be expected in this direction.

The writer stated the following in conclusion: If this remedy has any influence over the pelvic viscera which is directly due to an impression on the spinal cord, or upon the sympathetic ganglia, is doubtless a matter of some question, but it is highly probable that it has a tonic effect on the sympathetic ganglia, as well as the cerebro-spinal centers. He urged that the action of gelseminum over the disorders peculiar to women was a subject of great importance, and well worthy the attention of the profession. For a very large number of the pelvic disorders of women arise from a disturbed nervous system, which in turn produces morbid ovulation. It is in this way that I account for the action of gelseminum over these disorders. Its action is directly on the nerve-centers—spinal and sympathetic—equalizing the circulation and giving tone to cell power, and by these means permitting free ovulation, and relieving the congested and inflamed condition of the other pelvic viscera. If it does have this action, then Dr. Davis propounded the question, may it not also be considered a remedy for sterility?

The merits of the paper were discussed by Drs. G. C. Paoli, A. H. Foster, L. H. Montgomery, Robert Tilley and C. G. Davis, closing. These gentlemen's experiences and views accorded in many respects essentially with those reviewed by the essayist, although none of them had used it in so large a variety of cases. After which Dr. R. Tilley read a paper prepared by Dr. E. F. Ingals, giving a report of A Case of Nasal Catarrh Caused by a Large Cystic Tumor, with Removal and Recovery.

Mr. V. had suffered from catarrh more or less for the past ten years, and been especially troubled during the past six months. There was difficulty in breathing, and obstruction of the left nasal passage, being able to inhale with considerable exertion, and unable to exhale at all through that side. His hearing was also impaired. His general health, however, was excellent. Upon investigating the naso-pharynx by means of the rhinoscope, a smooth, grayish-white tumor was noticed, completely occluding the posterior orifice of the left nares, and filling two-thirds of the naso-pharyngeal cavity, which evidently sprang from the lateral wall of the pharynx in the region of the fossa of Rosen Mueller. An attempt was made to remove the growth with a modification of Jarvis' écraseur, armed with a fine steel piano wire (No. 5). After several trials, I found myself unable to pass the loop over the growth, on account of its great flexibility. A larger wire was then substituted, and aided by the rhinoscope, the growth was secured and finally detached. It fell into the mouth, and was ejected by the patient. It measured $1\frac{3}{8}$x$1\frac{1}{8}$ inches in the largest diameters, and $\frac{3}{4}$ of an inch in thickness. A microscopic examination of the tumor was made by Dr. E. P. Davis, who pronounced it to be a mucous cyst. No after-treatment was deemed necessary.

Careful examination of cases of so-called catarrh should be made, as many are due to nasal polypi, hypertrophied turbinated tissue, adenoid vegetation in the naso-pharynx, or tumors. A wax cast of the pathological specimen was exhibited, and remarks passed upon the growth of this variety of tumors, and what may be other fruitful causes of obstinate and obscure cases of catarrh.

The society, at a late hour, then adjourned.

